# Supplementary material for: Designer artificial environments for membrane protein synthesis
Source: Nat Commun. 2025 May 10;16:4363. doi: 10.1038/s41467-025-59471-1 (PMC12065789; doi:10.1038/s41467-025-59471-1)
Supplement: Supplementary file 1 — Supplementary Information [file 41467_2025_59471_MOESM1_ESM.pdf]

Designer artificial environments for membrane protein synthesis

Conary Meyer[1,+], Alessandra Arizzi[1,+], Tanner Henson[1, 2, 3], Sharon Aviran[1,4], Marjorie L. Longo[5], Aijun Wang[1, 2, 3], and Cheemeng Tan[1]*

[1]Department of Biomedical Engineering, University of California, Davis, Davis, CA 95616, USA

[2]Center for Surgical Bioengineering, Department of Surgery, University of California Davis School of Medicine, USA

[3]Institute for Pediatric Regenerative Medicine (IPRM), Shriners Children's Northern California, USA

[4]Genome Center, University of California, Davis, Davis, CA 95616, USA

[5]Department of Chemical Engineering, University of California, Davis, Davis, CA 95616, USA

*Correspondence to (cmtan@ucdavis.edu). [+]equal contribution

Supplementary Figure Legends

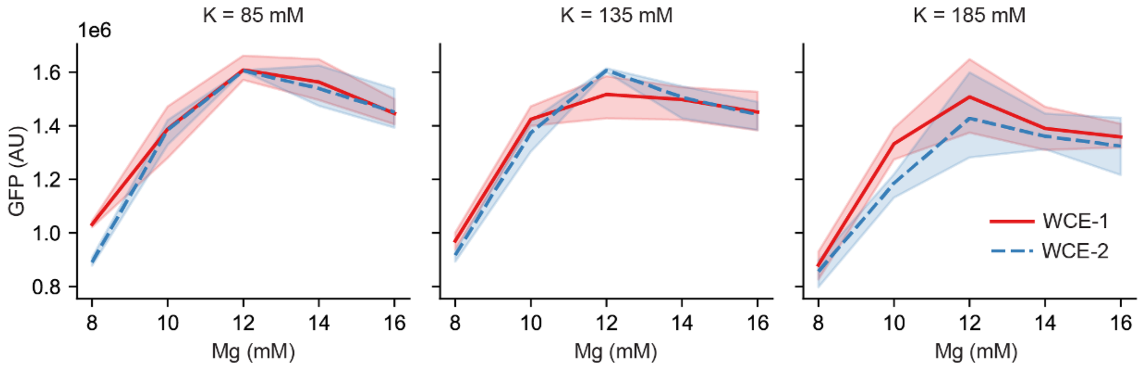

<u>Supplementary Figure 1: Benchmarking of different batches of whole cell extract.</u>

All CFPS are compared against each other across different concentrations of magnesium and potassium (n=3). The red solid lines represent one whole cell extract preparation, the blue dashed lines represent the next batch of whole cell extract. AU stands for Arbitrary Units and represents the direct measurement result from the plate reader. The average variance across all salt concentrations is 5.2%, with a maximal observed variance of 9.6% variance. The shaded area indicates the 95% confidence interval of the data. Source data are provided as a Source Data file.

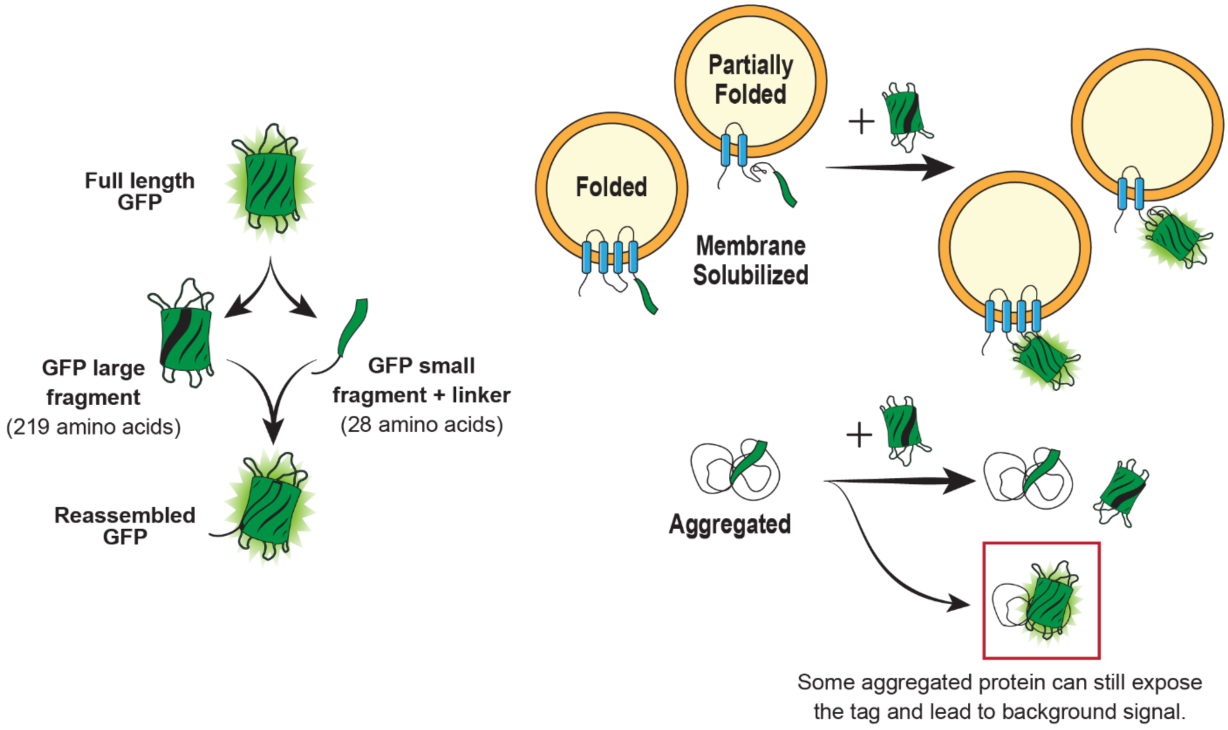

<u>Supplementary Figure 2: Schematic of the split-GFP solubilization reporter.</u>

The solubilization reporter utilizes two components. A large GFP fragment comprising the first 10 of the 11 beta strands of GFP and a small fragment containing the last beta-strand of GFP that can be attached to other proteins. Both components are independently non-fluorescent, but when mixed in the same reaction, they bind to one another and re-form the fluorophore.[5] The split-GFP reporter functions as a solubilization reporter because the binding of the two components is dependent on how accessible the small fragment is to bind the large fragment. If the synthesized protein is associated with the membrane, the C-terminally tagged GFP11 strand will likely be accessible to bind the large fragment and give fluorescence. It is possible for the membrane solubilized protein to be incorrectly folded but still present an exposed C-terminus, leading to a GFP signal. If the synthesized protein aggregates, the tag will be inaccessible and unable to bind the large fragment, showing minimal fluorescence. It is possible that the aggregated protein leaves the tag accessible for binding, leading to background fluorescence levels. This background fluorescence is captured in the no liposome control reactions that are subtracted out of all experimental reactions prior to analysis (Supplementary Note 1).

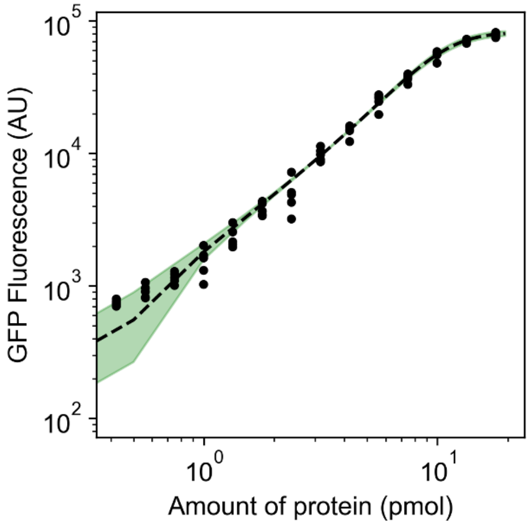

Supplementary Figure 3: Calibration curves based on purified protein with GFP11 tag are used to convert fluorescence intensity to protein quantity.

The calibration curve was generated using multiple dilutions of purified chloramphenicol acetyltransferase (Method 7) (n=5). The dashed line shows the sigmoid used to fit the data. The shaded green area indicates the 95% confidence interval of the model fit (Method 8). Source data are provided as a Source Data file.

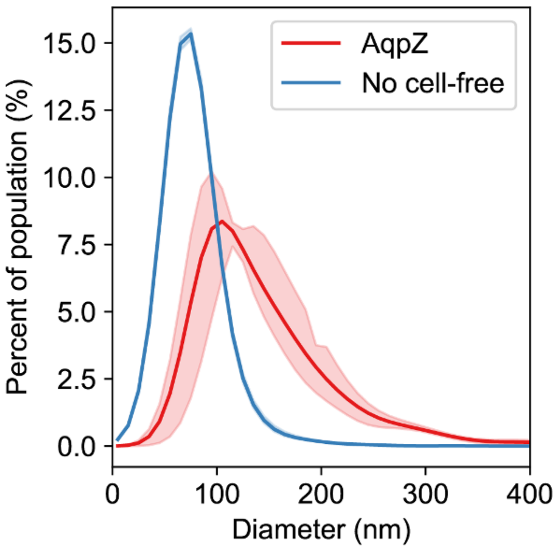

Supplementary Figure 4: Nanoparticle Tracking Analysis (NTA) shows monodisperse liposomes before cell-free protein synthesis and moderate liposome fusion post-synthesis.

The diameter of each identified liposome is measured, and the frequency of finding liposomes of a given diameter is shown. The blue line shows DOPC liposomes incubated overnight without cell-free system added. The red line shows DOPC liposomes incubated with the cell-free protein synthesis system producing AqpZ. The liposome-only samples show a median diameter of 75nm with a standard deviation of 15nm. The AqpZ-expressing samples show a median diameter of 115nm and a standard deviation of 43nm. The expression of AqpZ shows a 50% increase in the median diameter compared to the liposome-only samples ($p < 10^{-4}$). The shaded area in the plot represents the 95% confidence interval of the data (n=3). Source data are provided as a Source Data file.

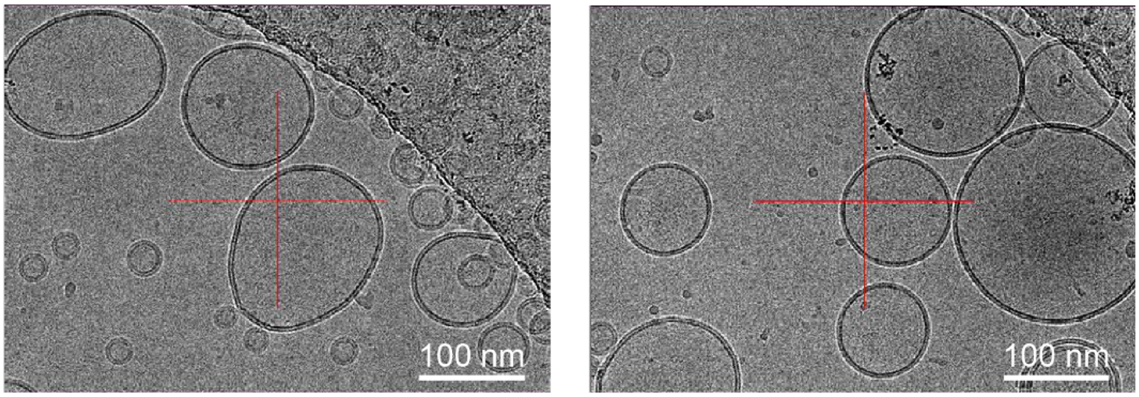

Supplementary Figure 5: Cryo-Electron microscopy images show unilamellar liposomes.

Cryo-EM images depict multiple liposomes generated using the standard liposome preparation procedure (Method 10). Micrographs were recorded at 56,818x (0.88 Å/pixel) with a dose of 40 e/A2. The scale bar indicates 100 nm in the image. Nearly all liposomes imaged are unilamellar (N=3 experiments).

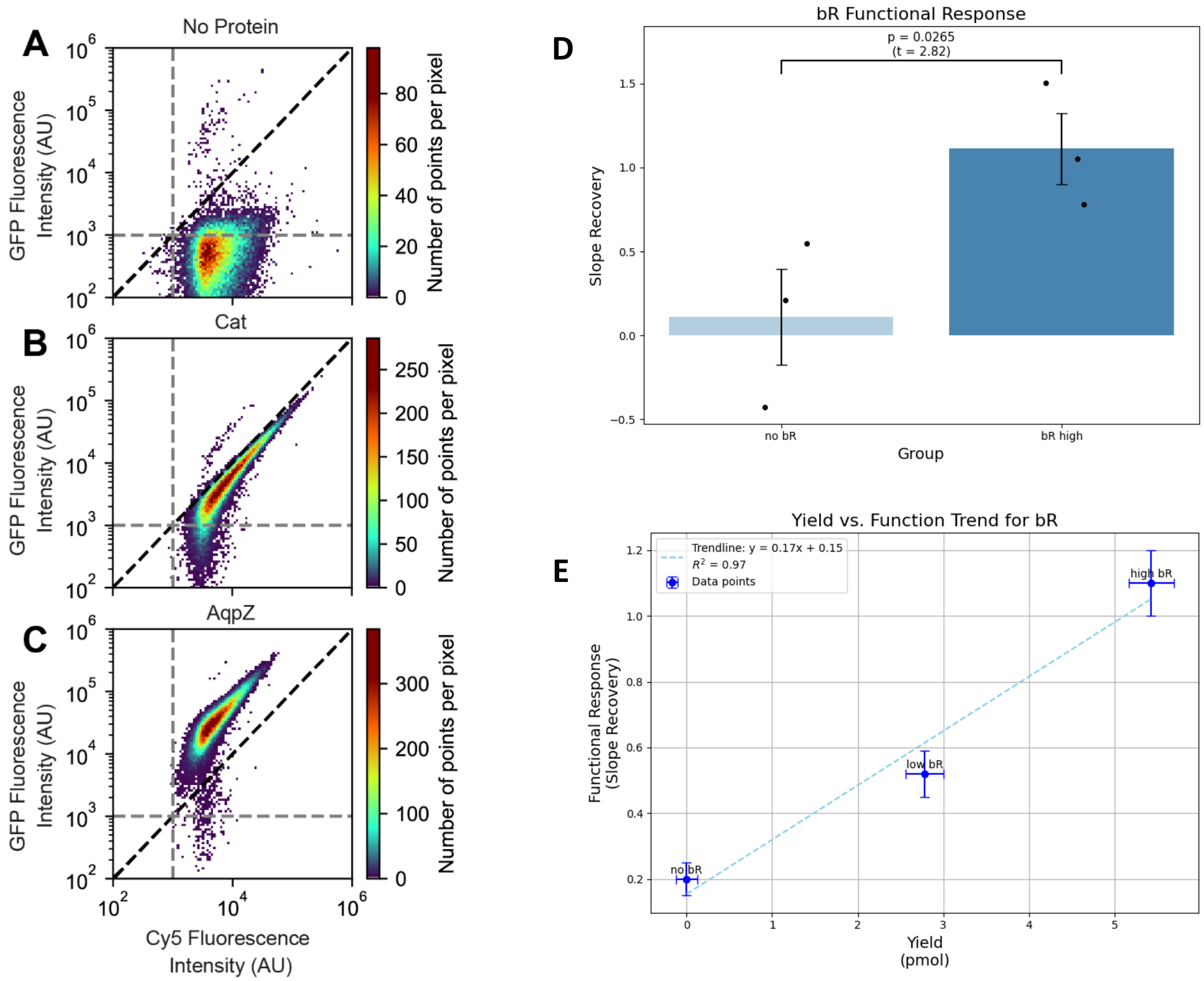

Supplementary Figure 6: Flow cytometry confirms AqpZ synthesis, and ACMA assay validates the correlation between split-GFP signal and protein activity.

(A-C) Density plots using the Red-660nm Area signal to monitor the Cy5 signal in the labeled liposomes and the Blue-525nm Area signal to monitor the split GFP report signal. Liposomes were produced with Cy5-labeled lipids included (Method 10) before adding them to the cell-free protein synthesis reaction. After the cell-free reaction was completed, and the large GFP fragment bound, samples were diluted and analyzed on the flow cytometer (Method 14). AU stands for Arbitrary Units, representing the signal values generated by the flow cytometer.

A) Samples with no DNA added to the cell-free protein synthesis reaction.

B) Samples with DNA encoding for Chloramphenicol Acetyltransferase (Cat) added to the cell-free protein synthesis reaction.

C) Samples with DNA encoding for expressing Aquaporin Z (AqpZ).

(D-E) The functional response of bR was tested using the ACMA assay, allowing quantitative measurements of ion flux across lipid membranes. Ion flux through a specific ion channel is measured via the varying fluorescence of the ACMA dye inside and outside the vesicles. In the case of bR, ion flux is caused by response to light. Source data are provided as a Source Data file.

D) The functional metric plotted here is calculated as the difference between the slope of the curve during the light response and the dark response (slope recovery). All plotted samples were normalized to the negative control (blank liposomes excluded from cell-free mixture). N=3 technical replicates, error bars represent SEM, one-sided t-test.

E) Correlation between split-GFP obtained yield and slope recovery. Each point represents the mean of n=3 technical replicates of slope recovery crossed with yield. Error bars represent the SEM.

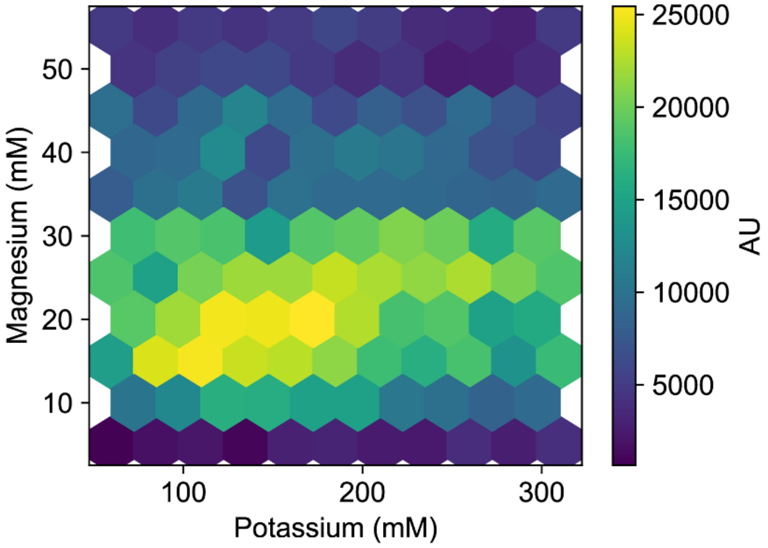

<u>Supplementary Figure 7: Cell-free protein synthesis shows broad salt tolerance.</u>

The general salt tolerance of CFPS was assessed by screening its ability to synthesize the test protein, deGFP, under various salt concentrations (n=3). All reactions tested with a Mg concentration of 5 mM showed, on average, only 7.2% of the fluorescence intensity seen for 15 mM. The ideal concentration of Mg for making deGFP was between 12.5 and 22.5 mM. However, we used 8 to 20mM in future experiments to provide a broader range of concentrations for membrane protein synthesis. The concentration limit for K was much broader than Mg, ranging from 85 to 185 mM. Source data are provided as a Source Data file.

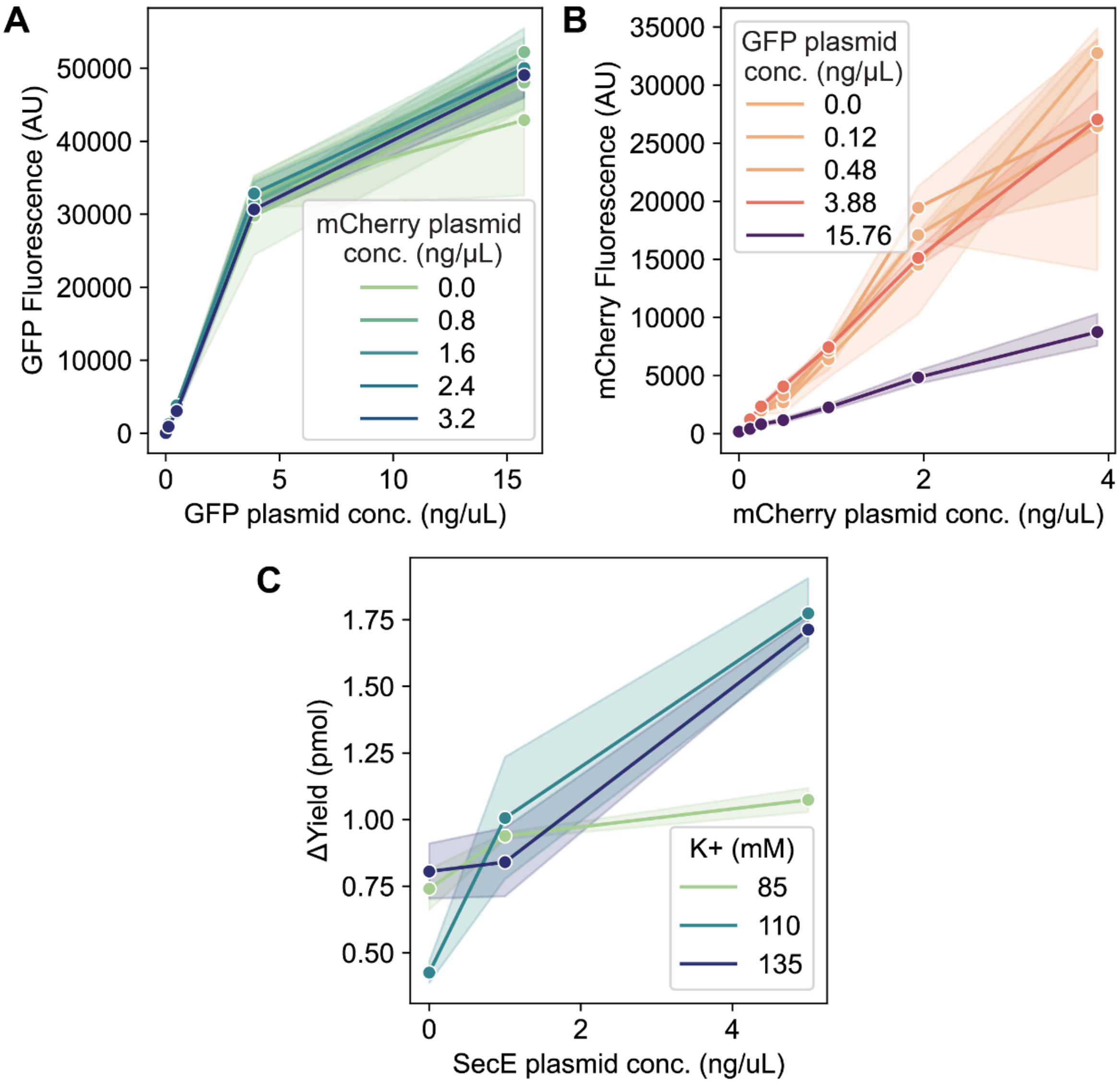

Supplementary Figure 8: Multiple proteins can be expressed simultaneously with minimal impact on one another at plasmid concentrations lower than 3 ng/µL.

Two plasmids encoding different proteins, deGFP and mCherry, were added into cell-free reactions at different concentrations to evaluate the impact of synthesizing two proteins at the same time. Plots show the change in protein fluorescence intensity as a function of the plasmid concentration in the CFPS reaction. Source data are provided as a Source Data file.

A) The observed GFP fluorescence intensity versus the amount of GFP plasmid added, stratified by the amount of mCherry plasmid in the reaction. The GFP fluorescence increases linearly with the GFP plasmid concentration up to 5 ng/µL. After 5 ng/µL of plasmid, the GFP fluorescence begins to saturate. Minimal impact from the concentration of the mCherry plasmid is observed, even up to 3 ng/µL. Shading represents the 95% confidence interval (n=3).

B) The observed mCherry fluorescence versus the amount of mCherry plasmid added, stratified by the amount of GFP plasmid in the reaction. The mCherry fluorescence increases linearly with the mCherry plasmid concentration for all tested concentrations. Minimal impact from the concentration of the GFP plasmid is observed, except when the GFP plasmid concentration is above 15 ng/µL. Shading represents the 95% confidence interval (n=3).

C) The observed split GFP signal from tagged SecY expression versus SecE plasmid concentration (n=3).

Source data are provided as a Source Data file.

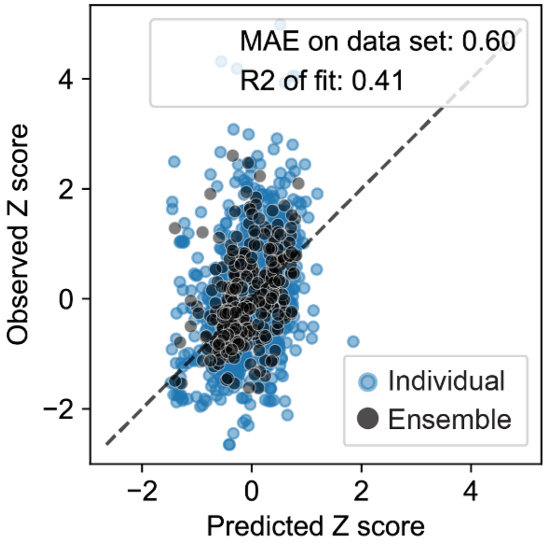

Supplementary Figure 9: Exclusion of protein identifiers greatly reduces model accuracy.

Predicted versus observed Z-scored values from an ensemble trained using only the reaction component concentrations on the screening data (Figure 2, Method 17). Blue: individual model predictions, Black: average of all model predictions versus average of all observed values from a specific reaction composition. The ensemble only achieves an R^2 value of 0.41. Hence, excluding the protein identifiers limits the model's ability to capture the protein-specific dependencies. Source data are provided as a Source Data file.

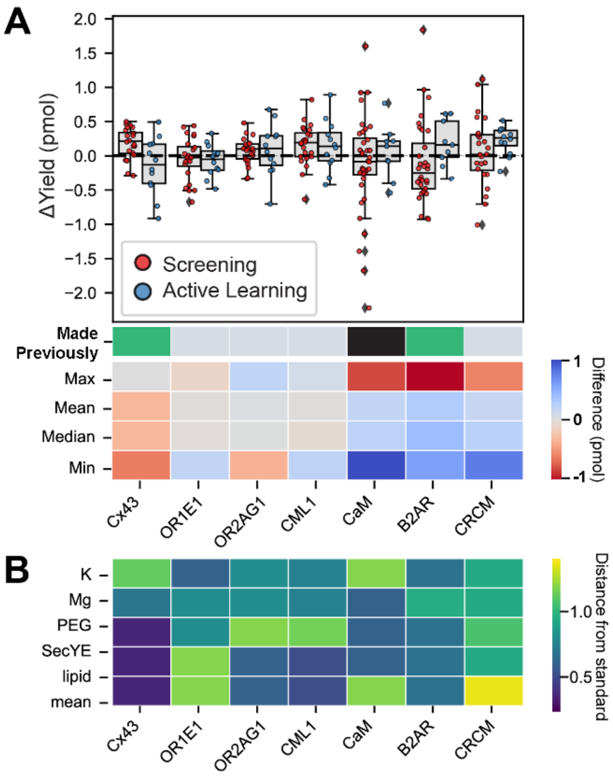

Supplementary Figure 10: Some proteins did not benefit from the active learning reaction selection.

A) Comparison of the protein yield between the screening reactions (red points) and the active learning selected reactions (blue points). The points represent the average of replicates (n≥3) for a given reaction composition. All proteins in this plot showed no statistically significant (t-test p-value > 0.05) difference between the screening and active learning reactions. The bar immediately below the box plots indicates whether the protein has been reportedly attempted in previous works. Grey indicates it has not been reported. Black indicates it has been tried but was unsuccessful in cell-free protein synthesis using liposomes. Green indicates that it was shown to be successful. The heat map below indicates the changes between the screening and active learning populations.

B) The Euclidean norm is calculated using differences between each reaction component's concentration in the standard and the optimal concentration and the resulting yield in the standard and optimal concentration.

Source data are provided as a Source Data file.

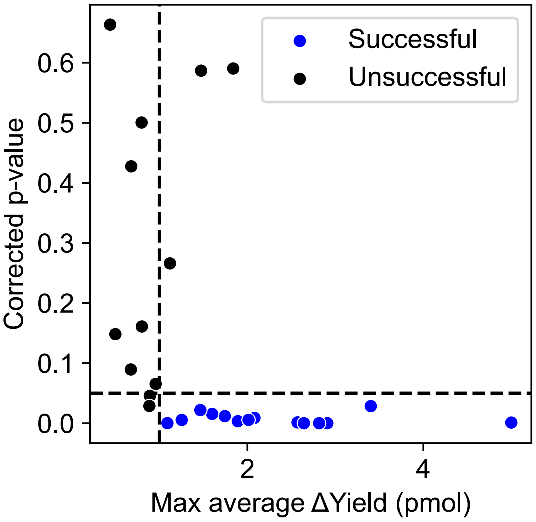

Supplementary Figure 11: Separation between reactions labeled as successful versus unsuccessful.

Scatterplot showing the reactions with the highest average change in yield for each protein plotted against the p-value corrected using the Benjamini–Hochberg method (Method 21). These reactions include all conditions tested in both the screening and active learning reactions (Figure 3C and Supplementary Figure 10). The max average yield was clipped to 5 pmol to better illustrate the separation of the data. The vertical dashed line at 1 pmol and horizontal line at 0.05 show the cutoffs for "successful" synthesis. 12 proteins were labeled as unsuccessful, and the remaining 16 as successful. Source data are provided as a Source Data file.

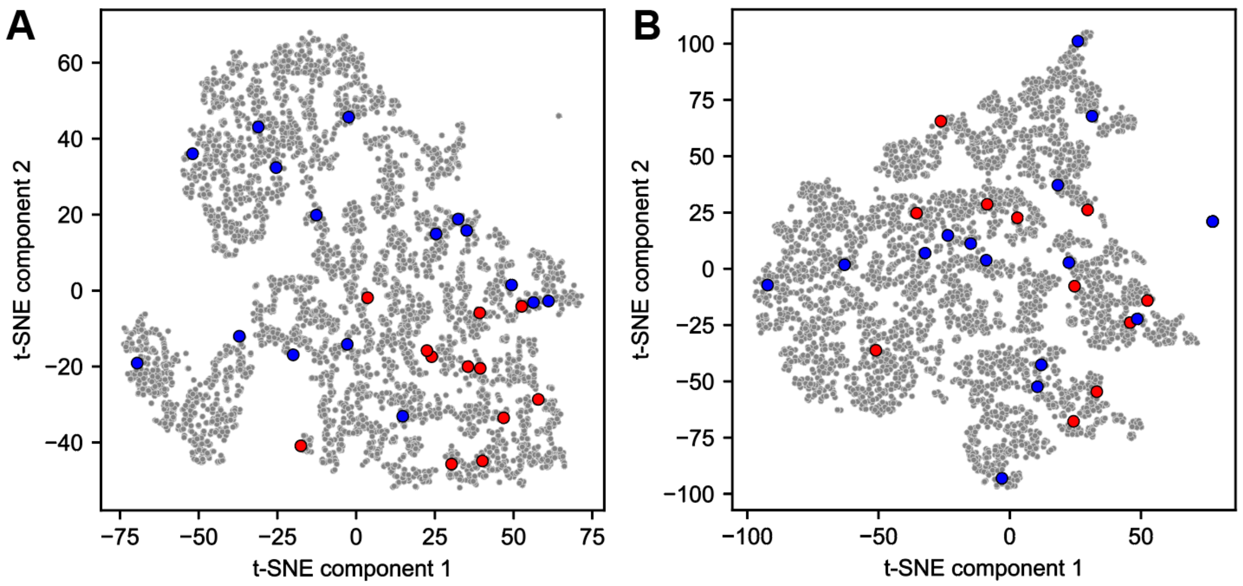

Supplementary Figure 12: Successfully and unsuccessfully membrane-incorporated proteins show different grouping in different embedded spaces.

The tested proteins are plotted with the other proteins in the structure dataset in different embedded spaces generated from different subsets of the protein structure features (Methods 21 and 22). Blue markers are successfully membrane-incorporated proteins (n=16), red markers are unsuccessfully membrane-incorporated proteins (n=12), and grey markers are all other proteins in the dataset (n=4584).

A) The tSNE embedded space was generated using the 4 metrics (charge, depth, hydrophobicity, and probability) of the amino acids located in the inner hydrophobic layer in contact with the membrane. The two classes of proteins appear to separate well from one another, suggesting that the features included in the embedding relate to a protein's ability to be synthesized in this system.

B) The tSNE embedded space was generated using the 4 metrics (charge, depth, hydrophobicity, and probability) of the amino acids located in the water layer outside the membrane. The two classes appear to be mixed together, suggesting that the features included in the embedding are not relevant to a protein's ability to be incorporated into liposomes using this system.

Source data are provided as a Source Data file.

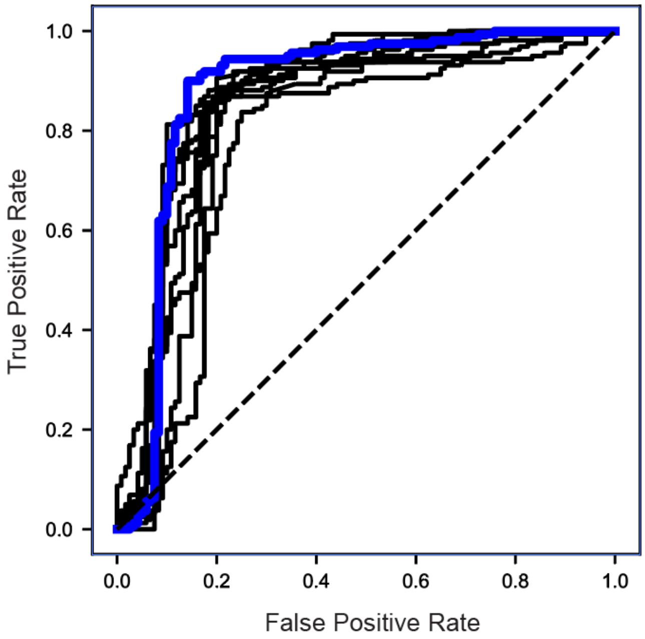

Supplementary Figure 13: Ensemble Classifiers achieve high accuracy predictions on test data.

The Receiver Operating Characteristic (ROC) curves for the 10 Ensemble Classifiers with accuracy above 83%. The results show that the classifiers outperform random guessing. Source data are provided as a Source Data file.

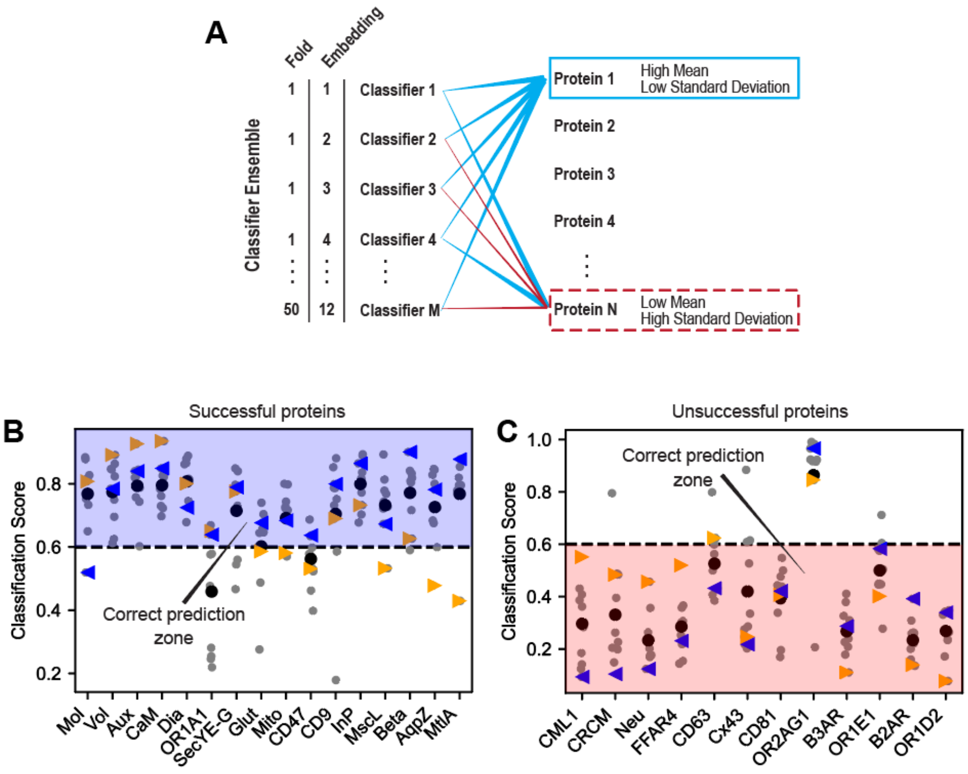

Supplementary Figure 14: The Ensemble Classifiers can accurately predict the success or failure of membrane protein synthesis unseen during training.

A) Illustration of the ensemble-based classification. 600 different SVM classifiers were trained using: 1) 50 different train:test splits 2) The 10 paired embeddings with an accuracy of greater than 83%. The Ensemble Classifiers are then used to predict the synthesis success of unseen proteins. The reactions with consistently high predicted values are presumed to be more likely to be synthesized (Method 22).

B) Predictions from the Ensemble Classifiers on all successfully made proteins that were removed from each model's training data. Proteins that score above 0.6 are correctly labeled.

C) Predictions from the Ensemble Classifiers on all unsuccessfully made proteins that were removed from each model's training data. Proteins that score below 0.6 are correctly labeled.

Grey points represent the average predicted score for all Ensemble Classifiers trained on the same embeddings. Black points represent the average of all Ensemble Classifier predictions for that specific protein. The triangles are included as a reference for the differences between each Ensemble Classifier's performance. Orange triangles are the model's most accurate predictions,

blue triangles are the predictions for the least accurate model in the included set. The dashed line indicates the average decision boundary used by the classifiers to assign the binary label. Differing model predictions indicate uncertainty and provide a confidence metric for assessing the ensemble's predictions.

Source data are provided as a Source Data file.

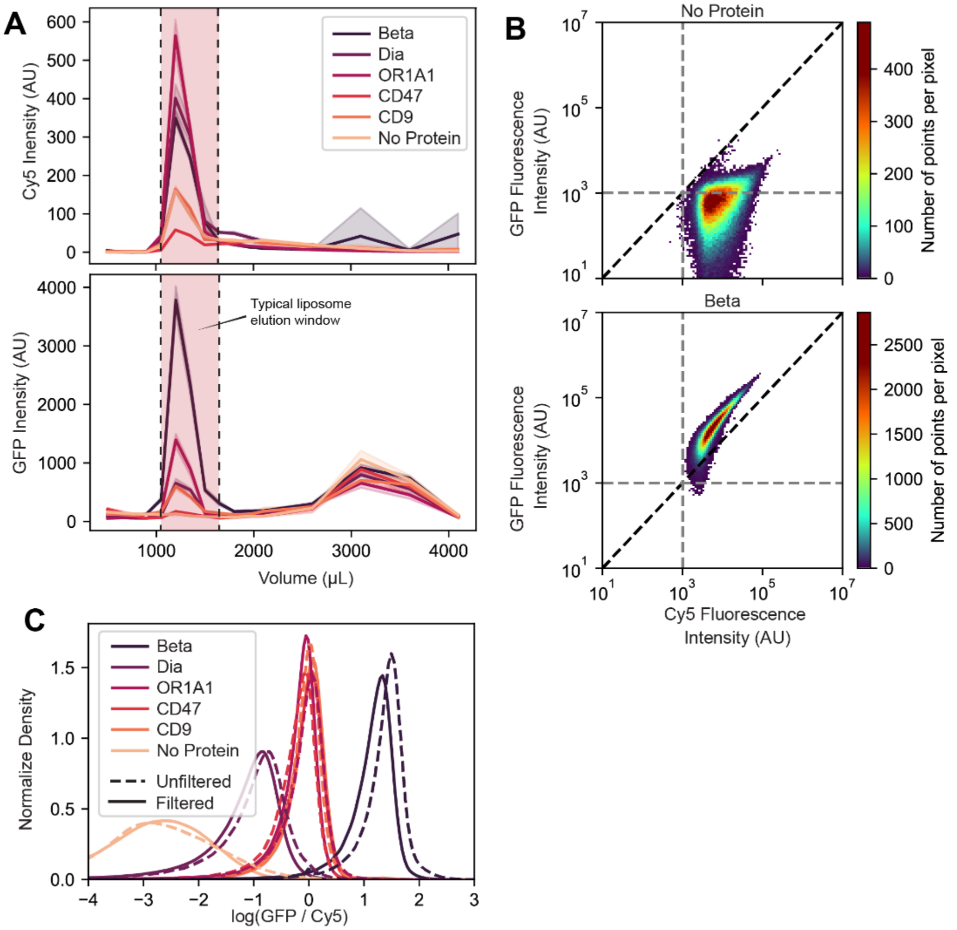

Supplementary Figure 15: Additional results show membrane proteins colocalize with liposomes.

A) All three proteins show an increase in GFP signal in the same elution volume as the liposomes during Size-Exclusion Chromatography (SEC) (Method 13). Top panel: Liposome elution monitoring Cy5 signal from labeled lipids. Bottom panel: GFP elution monitoring GFP reporter bound to liposomes/membrane proteins. Red shaded area indicates the expected elutions for liposomes. No protein samples had no plasmid DNA added at the beginning of the cell-free reaction. (n=3, 3 individually prepared liposome populations were used for all 3 proteins, so all proteins were tested using the same liposome samples)

B) Density plots using the Red-660nm Area signal to monitor the Cy5 signal in the labeled liposomes and the Blue-525nm Area signal to monitor the split GFP report signal. Liposomes were produced with Cy5-labeled lipids included (Method 10), before adding them to the cell-free

protein synthesis reaction. After the cell-free reaction was completed, and the large GFP fragment had bound, samples were diluted and analyzed on the flow cytometer (Method 14). AU stands for Arbitrary Units, representing the signal values generated by the flow cytometer. Top panel: Samples with no plasmid DNA added to the cell-free protein synthesis reaction. Bottom panel: Samples with DNA encoding for Beta added to the cell-free protein synthesis reaction. (n=3, 100,000 counts each).

C) Kernel density plot shows the distribution of the log(GFP/Cy5) values taken from events recorded during flow cytometry (Method 14). No protein-liposomes show far lower ratios than the other membrane proteins. (flow cytometry samples n=3, 100,000 events recorded for each). Dashed lines represent samples prior to SEC, solid lines represent samples after SEC. (n=3).

Source data are provided as a Source Data file.

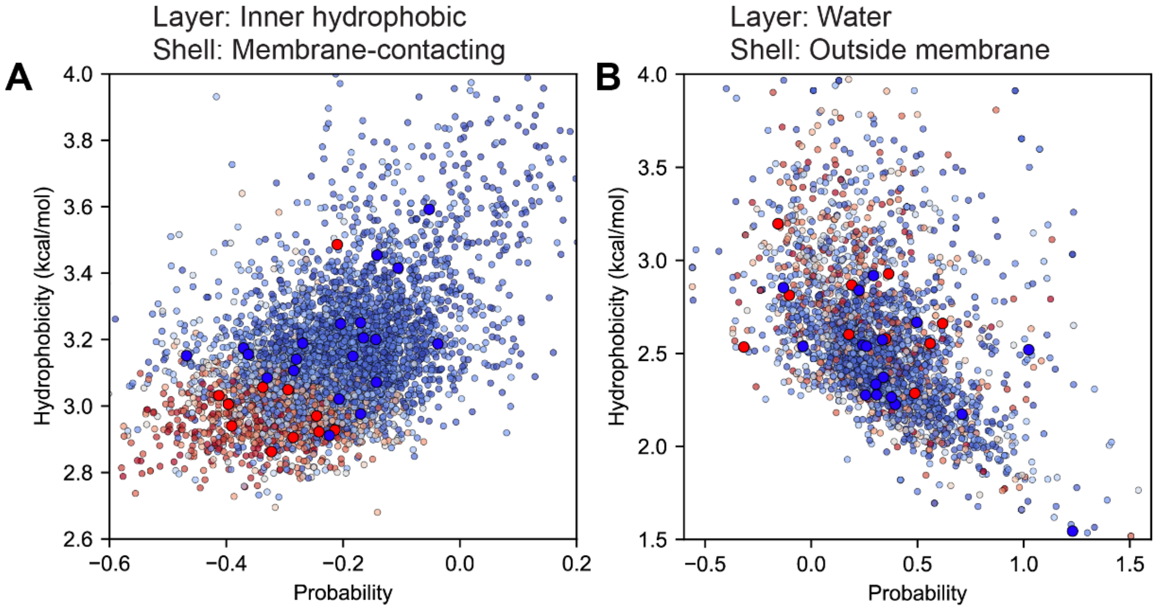

Supplementary Figure 16: Chemical features associated with membrane-contacting amino acids in the inner hydrophobic layer can help to classify successful and unsuccessful proteins.

The hydrophobicity and probability values plotted represent the average hydrophobicity and probability values for all amino acids in each protein in

A) The inner hydrophobic layer and the membrane-contacting shell.

B) The water layer and the outside membrane shell.

Large points indicate that the protein was experimentally tested (n=28) (Red points were not successfully made, blue were successfully made). Small points indicate proteins that were not tested, but the likelihood of successful synthesis was predicted by the Ensemble Classifiers (n=4,584, Method 22) (The hue indicates the predicted outcome of synthesis: red points were predicted to not be successfully made, blue were predicted to be successfully made).

Source data are provided as a Source Data file.

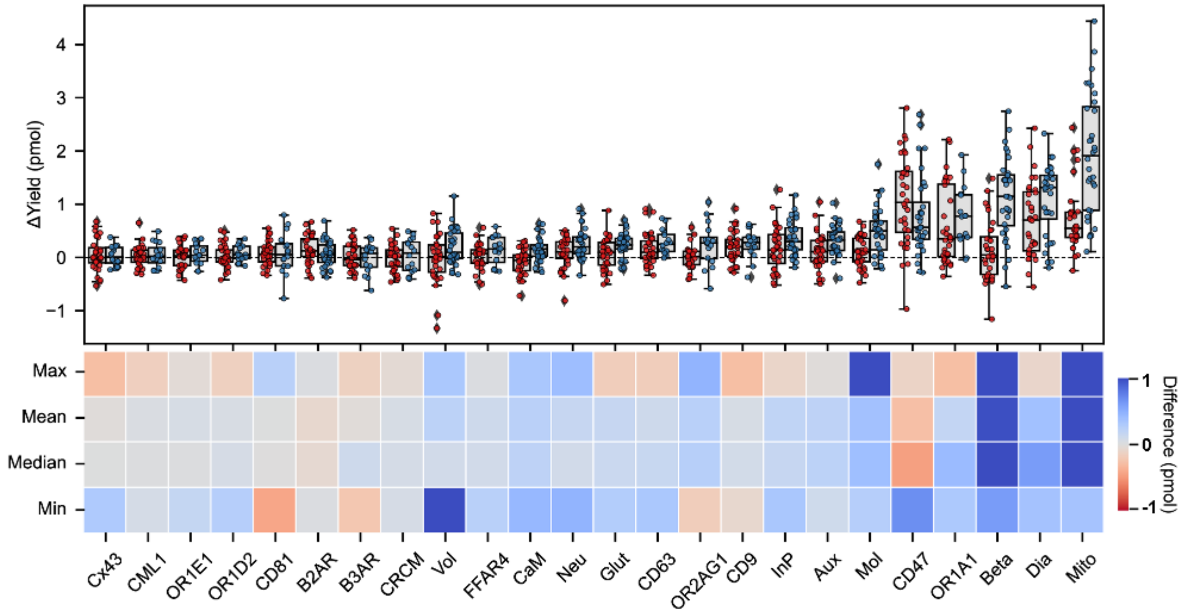

Supplementary Figure 17: Expanding the experimental search space can be detrimental to active learning performance.

The points represent the average of all replicates for a given reaction composition. The screening (red dots) and active learning (light blue dots) reactions are shown for each protein (n≥48 unique compositions per protein, 4 with liposome and 4 without liposome reactions per composition). The heat map below indicates the changes (max, mean, min, and median) between the screening and active learning populations. Source data are provided as a Source Data file.

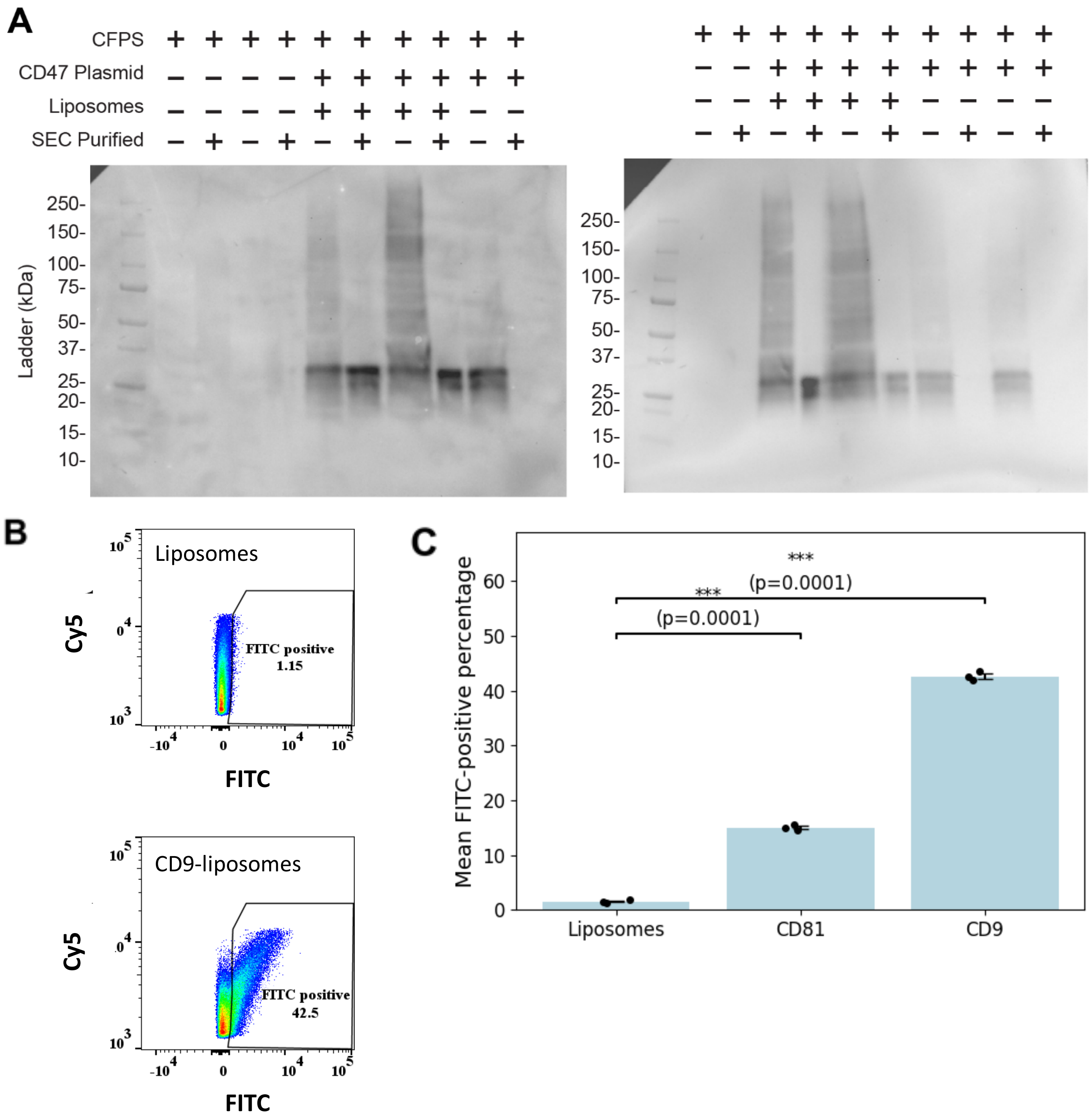
A
CFPS
CD47 Plasmid
Liposomes
SEC Purified
Ladder (kDa)
250-
150-
100-
75-
50-
37-
25-
20-
15-
10-
B
Liposomes
Cy5
FITC positive
1.15
FITC
CD9-liposomes
FITC positive
42.5
C
Mean FITC-positive percentage
***
(p=0.0001)
***
(p=0.0001)
Liposomes
CD81
CD9

Supplementary Figure 18: Leukocyte surface antigen CD47 expression and membrane association is confirmed by Western blot, and CD9 and CD81 have exposed functional domains detectable by flow cytometry.

A) Three cell-free synthesis reactions were tested: 1) a negative control with only CFPS, 2) CFPS with the plasmid encoding CD47 added to the reaction but excluding liposomes, and 3) CFPS with both the CD47 plasmid and liposomes added. Samples from the reactions were taken before and after size-exclusion chromatography (Method 13). The primary antibody used in the Western blot targets CD47 directly (Method 23). No bands were observed in the reactions that included only CFPS indicating minimal off-target binding. Bands were observed in all reactions that included the CD47 plasmid. The most prominent band was located near the 37 kDa band in the ladder, indicating that the full-length CD47 protein (35.2 kDa) was synthesized. When the reactions without liposomes were purified by size exclusion chromatography, no band was observed. However, samples with liposomes showed a clear band of the target size after SEC indicating that the protein was associated with the liposomes (n=3 experimental replicates).

B) Flow cytometry analysis using an antibody that is specific to an extracellular domain of CD9. The top panel shows minimal signal in the FITC channel, indicating little observable anti-CD9 binding in the sample containing blank liposomes incubated in a cell-free mixture. The lower panel shows the data obtained from samples expressing CD9. (n=3 technical replicates, unpaired t-test).

C) Quantification of CD9- and CD81-bearing liposomes relative to blank liposome controls incubated in cell-free mixture. N=3 technical replicates for all groups, $p<0.001$=***, calculated with Welch's t-test and critical values from the studentized range distribution ($k = 3$, $\alpha = 0.05$). Error bars represent SEM.

Source data are provided as a Source Data file.

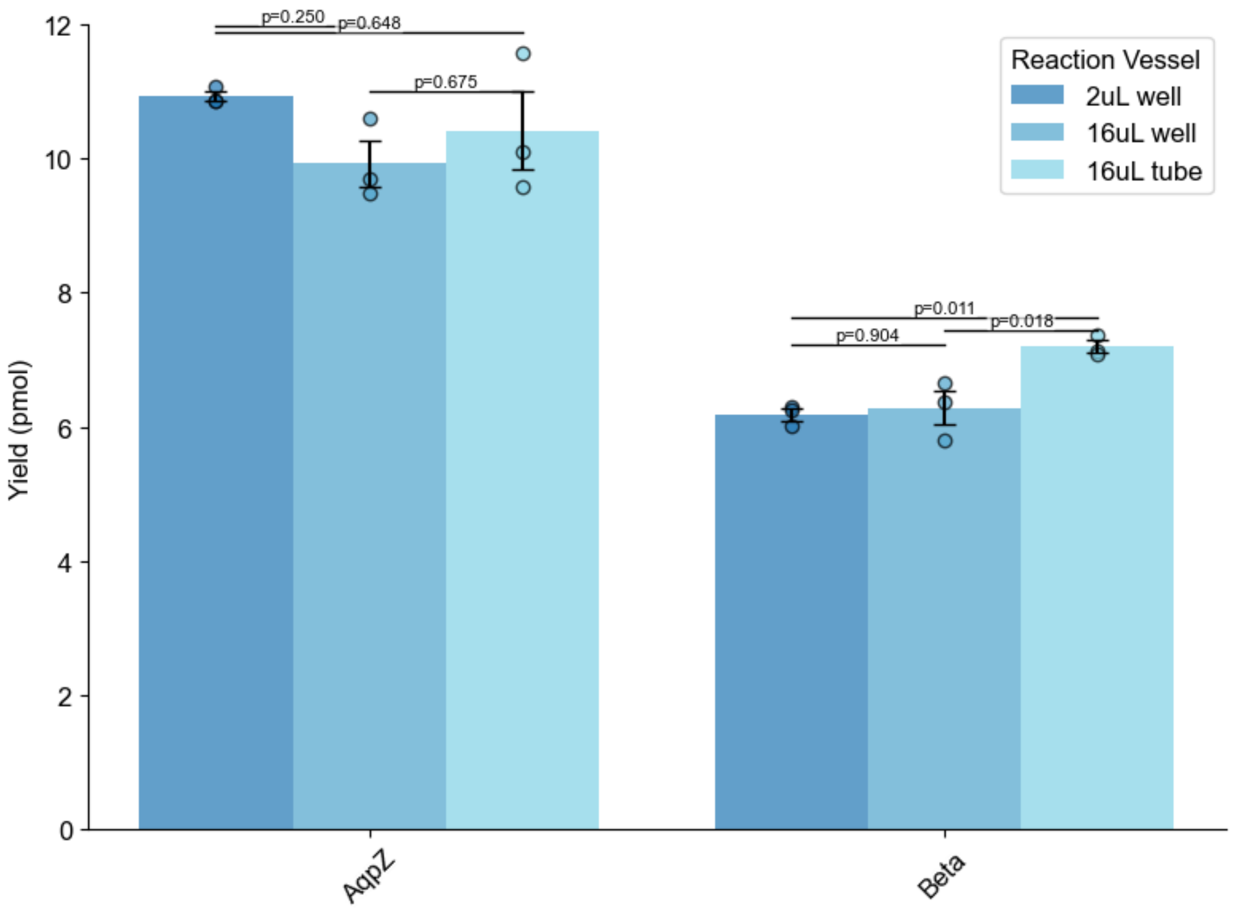

Supplementary Figure 19: Volume and reaction vessel have minimal effect on protein production,

The chosen reaction compositions used for the two tested proteins, AqpZ and Beta, were evaluated across two different reaction volumes and vessels. The standard reaction type used in all previously reported data was 2µL in a 384-well plate. The additional conditions included the same well plate but with reaction volumes of 16 µL, and 16µL reaction in microfuge tubes. (p-values calculated using TukeyHSD, n=3, $\alpha = 0.05$). Error bars represent SEM. Source data are provided as a Source Data file.

Supplementary Notes

Supplementary Note 1: Negative control reactions were used to evaluate the amount of background signal from aggregated protein.

Every tested reaction had a negative control reaction with the exact same composition, excluding the liposomes. This negative control represented the upper bound of the signal that would be obtained if all the produced proteins aggregated. The success of a given reaction was assessed using the observed signal in the "without liposome" reaction subtracted from the "with liposome" reaction.

Supplementary Note 2: The split-GFP reporter quantifies membrane solubilization of cell-free synthesized membrane proteins.

Cell-free synthesized membrane proteins, such as most GPCRs, transporters, or channels synthesized in this work, associate with the membrane during mRNA translation. However, not all of the membrane-associated proteins fold into fully functional conformations. Only a fraction of membrane-solubilized GPCR may be folded correctly depending on the protein being synthesized[20]. A key challenge of any protein synthesis study is the lack of assay or technology to quantify these partially folded, non-functional protein fractions. The fluorescence of the complemented split-GFP reporter measures primarily the amount of membrane solubilized membrane protein. For any given membrane protein, the split-GFP solubilization reporter may correlate with a certain functional fraction of membrane protein. MEMPLEX does not include a testing step for functional vs non-functional membrane proteins. Supplementary Figure 6 shows an example of this test using Bacteriorhodopsin (bR).

Supplementary Note 3: Using calibration curve to convert split-GFP fluorescence to protein quantity.

A calibration curve was measured during every experiment to compensate for the observed variability between batches of the large GFP fragment, which must be solubilized and filtered fresh before every experiment. The curve was generated by binding the large fragment to different concentrations of the purified soluble protein Cat tagged with the small GFP fragment. The curve deviates from linearity at protein amount above 15 pmol. For this reason, a sigmoidal

function is used to fit the calibration curve used in each experiment (Supplementary Figure 3, Methods 7 and 8).

Supplementary Note 4: Topology limitations of the split GFP complementation-based reporter system.

The split GFP complementation assay requires that the GFP11 tag and the large GFP1-10 fragment be on the same side of the membrane. As the large fragment is supplemented to the reactions, it is located outside of the membrane. This also requires the tag to be located on the outside of the membrane. The proteins are synthesized and inserted from the outside of the membrane, thereby reversing the orientation of the synthesized protein, assuming that it maintains its native orientation. Therefore, the cytoplasmic regions of the protein would be located outside the membrane and available to interact with the large fragment. All proteins included in the set with known orientation have a cytoplasmic C-terminus, which allows for the detection of the protein using this assay. It is unclear whether the original orientation of the protein is maintained during spontaneous insertion. Some work indicates that the synthesized protein is randomly oriented (Periasamy et al. 2013) while others indicate that proteins strongly favor the original orientation (Wuu et al. 2008, Wanzer et al. 2021). The yields reported in this work only capture the proteins that are oriented with the C-terminus facing outward.

Supplementary Note 5: Motivation behind the reaction parameter selection.

The cations potassium and magnesium glutamate are commonly optimized parameters in cell-free protein synthesis as they help mediate translation and stabilize RNA secondary structure. These parameters are also important in the context of membrane protein synthesis because they can interact with lipids, causing changes in the hydration state and overall packing of the lipids[21]. Furthermore, they have also been shown to directly impact the insertion rates of cell-free expressed proteins[22]. PEG8000 is commonly used in cell-free protein synthesis reactions as a molecular crowding agent. By forcing all the translation components near one another, it can increase translation rates[23]. In addition to this role, PEG8000 can also interact directly with the surface of liposomes[24]. This interaction could interfere with the nascent membrane protein's ability to interact with the membrane and decrease insertion rates. The lipids that were chosen

differed in their hydrocarbon tail lengths. The change in tail length changes several properties, such as the length of the hydrophobic section of the membrane and the fluidity of the membrane. Each protein will have a different hydrophobic length that needs to match the lipids and the change in fluidity could help certain proteins insert into the membrane. The inclusion of the plasmid sequences for SecY and SecE as a variable instead of always including them is because the expression of additional proteins in the cell-free reaction will divert resources away from the target protein. Even though the inclusion of the translocon should be helpful, its production could be harmful.

Supplementary Note 6: Defining the concentration ranges for all reaction components.

The concentration range of magnesium and potassium glutamate was determined by conducting a broad salt screen on the synthesis of GFP (Supplementary Figure 7). The concentration of PEG 8000 was set between 0-2%, which is the standard concentration our lab uses and the highest typically seen in other work. The concentration of the SecY and SecE plasmids was set based on the limit of multiple protein expression, setting the high concentration below the concentration where there is a clear detriment to the synthesis of the other proteins (Supplementary Figure 8). The lipid types chosen provide a range of membrane thicknesses, with DMPC having 14-carbon tails and DOPC with 18-carbon tails.

Supplementary Tables

Supplementary Table 1: Reaction compositions in Figure 1B:

Reaction Number	Lipid Type	Mg (mM)	SecYE (ng/uL)	K (mM)	PEG (%)
1	DMPC	8	0	85	2
2	DMPC	8	1.25	85	2
3	DOPC	8	0	85	2
4	DOPC	8	0	185	2

Supplementary Table 2: Proteins tested in this work.

Uniprot ID	Short name	Full name	Organism	Length (amino acids)	Transmembrane Domains	Mass (kDa)
P60844	AqpZ	Aquaporin Z	E. coli	231	6	23.7
F4HWB6	Aux	Auxin efflux carrier-like protein 1	A. thaliana	472	10	51.9
P07550	B2AR	Beta-2 adrenergic receptor	H. sapiens	413	7	46.5
P13945	B3AR	Beta-3 adrenergic receptor	H. sapiens	408	7	43.5
Q9LTG0	Beta	Beta-carotene 3-hydroxylase 2	A. thaliana	303	4	33.8
Q8IU99	CaM	Calcium homeostasis modulator protein 1	H. sapiens	346	4	38.3
Q08722	CD47	Leukocyte surface antigen CD47	H. sapiens	323	4	35.2
P08962	CD63	CD63 antigen	H. sapiens	238	4	25.6
P60033	CD81	CD81 antigen	H. sapiens	236	4	25.8
P21926	CD9	CD9 antigen	H. sapiens	228	4	25.4
Q99788	CML1	Chemerin-like receptor 1	H. sapiens	373	7	42.3
Q96D31	CRCM	Calcium release-activated calcium channel protein 1	H. sapiens	301	4	32.7
P17302	Cx43	Connexin-43	H. sapiens	382	4	43.0
Q8L4R0	Dia	Protein Trigalactosyl-Diacylglycerol Transporter	A. thaliana	350	6	37.9
Q5NUL3	FFAR4	Free Fatty Acid Receptor 4	H. sapiens	361	7	40.5
Q7Z429	Glut	Glutamate [NMDA] receptor-associated protein 1	H. sapiens	371	7	41.2
Q38954	InP	Inorganic phosphate transporter 2	A. thaliana	587	12	61.4
Q9UDX5	Mito	Mitochondrial fission process protein 1	H. sapiens	166	3	18.0
Q9SL95	Mol	Molybdate transporter 1	A. thaliana	456	9	48.4
P0A742	MscL	Large-conductance mechanosensitive channel	E. coli	136	2	15.0
P00550	MtlA	Mannitol permease IIC component	E. coli	637	6	68.0
Q9BWQ8	Neu	Neural membrane protein 35	H. sapiens	316	7	35.1
Q9P1Q5	OR1A1	Olfactory receptor 1A1	H. sapiens	309	7	34.6
P34982	OR1D2	Olfactory receptor 1D2	H. sapiens	312	7	35.2
P30953	OR1E1	Olfactory receptor 1E1	H. sapiens	314	7	35.3
Q9H205	OR2AG1	Olfactory receptor 2AG1	H. sapiens	316	7	35.3
P0AGA2	SecYE-G	Protein translocase subunit SecY	E. coli	443	12	48.5
Q5JW98	Vol	Calcium homeostasis modulator protein 4	H. sapiens	314	4	35.1

Supplementary References

1. Weber, E., Engler, C., Gruetzner, R., Werner, S. & Marillonnet, S. A Modular Cloning System for Standardized Assembly of Multigene Constructs. *PLOS ONE* **6**, e16765 (2011).
2. Moore, S. J. *et al.* EcoFlex: A Multifunctional MoClo Kit for E. coli Synthetic Biology. *ACS Synth. Biol.* **5**, 1059–1069 (2016).
3. Meyer, C. *et al.* High-Throughput Experimentation Using Cell-Free Protein SynthesisCell-free protein synthesis (CFPS) Systems. in *Cell-Free Gene Expression: Methods and Protocols* (eds. Karim, A. S. & Jewett, M. C.) 121–134 (Springer US, New York, NY, 2022). doi:10.1007/978-1-0716-1998-8_7.
4. Wang, J. *et al.* Microfluidic cap-to-dispense (μCD): a universal microfluidic–robotic interface for automated pipette-free high-precision liquid handling. *Lab. Chip* **19**, 3405–3415 (2019).
5. Cabantous, S. & Waldo, G. S. In vivo and in vitro protein solubility assays using split GFP. *Nat. Methods* **3**, 845–854 (2006).
6. Virtanen, P. *et al.* SciPy 1.0: fundamental algorithms for scientific computing in Python. *Nat. Methods* **17**, 261–272 (2020).
7. Cossarizza, A. *et al.* Guidelines for the use of flow cytometry and cell sorting in immunological studies (second edition). *Eur. J. Immunol.* **49**, 1457–1973 (2019).
8. *Flow Cytometry Protocols*. vol. 1678 (Springer New York, New York, NY, 2018).
9. Surowiec, I. *et al.* Generalized Subset Designs in Analytical Chemistry. *ACS Publications* https://pubs.acs.org/doi/epdf/10.1021/acs.analchem.7b00506 (2017) doi:10.1021/acs.analchem.7b00506.
10. Seabold, S. & Perktold, J. Statsmodels: Econometric and Statistical Modeling with Python. in 92–96 (Austin, Texas, 2010). doi:10.25080/Majora-92bf1922-011.

11. Abadi, M. *et al.* TensorFlow: Large-Scale Machine Learning on Heterogeneous Distributed Systems.

12. Pedregosa, F. *et al.* Scikit-learn: Machine Learning in Python. *Mach. Learn. PYTHON.*

13. The UniProt Consortium. UniProt: the Universal Protein Knowledgebase in 2023. *Nucleic Acids Res.* **51**, D523–D531 (2023).

14. Jumper, J. *et al.* Highly accurate protein structure prediction with AlphaFold. *Nature* **596**, 583–589 (2021).

15. Varadi, M. *et al.* AlphaFold Protein Structure Database: massively expanding the structural coverage of protein-sequence space with high-accuracy models. *Nucleic Acids Res.* **50**, D439–D444 (2022).

16. Yarov-Yarovoy, V., Schonbrun, J. & Baker, D. Multipass membrane protein structure prediction using Rosetta. *Proteins Struct. Funct. Bioinforma.* **62**, 1010–1025 (2006).

17. Edelsbrunner, H. & Mücke, E. P. Three-dimensional alpha shapes. *ACM Trans Graph* **13**, 43–72 (1994).

18. Wimley, W. C. & White, S. H. Experimentally determined hydrophobicity scale for proteins at membrane interfaces. *Nat. Struct. Biol.* **3**, 842–848 (1996).

19. Buitinck, L. *et al.* API design for machine learning software: experiences from the scikit-learn project. Preprint at http://arxiv.org/abs/1309.0238 (2013).

20. Grisshammer, R. & Tateu, C. G. Overexpression of integral membrane proteins for structural studies. *Q. Rev. Biophys.* **28**, 315–422 (1995).

21. Binder, H. & Zschörnig, O. The effect of metal cations on the phase behavior and hydration characteristics of phospholipid membranes. *Chem. Phys. Lipids* **115**, 39–61 (2002).

22. Altrichter, S., Haase, M., Loh, B., Kuhn, A. & Leptihn, S. Mechanism of the Spontaneous and Directional Membrane Insertion of a 2-Transmembrane Ion Channel. *ACS Chem. Biol.* **12**, 380–388 (2017).

23. Tan, C., Saurabh, S., Bruchez, M. P., Schwartz, R. & LeDuc, P. Molecular crowding shapes gene expression in synthetic cellular nanosystems. *Nat. Nanotechnol.* **8**, 602–608 (2013).

24. Mishima, K., Satoh, K. & Suzuki, K. Increase in molecular order of phospholipid membranes due to osmotic stress by polyethylene glycol. *Colloids Surf. B Biointerfaces* **10**, 113–117 (1997).
