# Supplementary material for: Designer artificial environments for membrane protein synthesis
Source: Nat Commun. 2025 May 10;16:4363. doi: 10.1038/s41467-025-59471-1 (PMC12065789; doi:10.1038/s41467-025-59471-1)
Supplement: Supplementary file 2 — Description of Additional Supplementary Files [file 41467_2025_59471_MOESM2_ESM.pdf]

Description of Additional Supplementary Files:

Supplementary Data 1: DNA sequences of proteins. List of complete DNA sequences for each construct is provided here. This workbook provides the complete nucleotide sequences and annotation for every plasmid construct used in this study. Each row is named for the corresponding protein (short name) and includes: membrane protein coding sequences, additional membrane protein expression sequences, additional protein sequences, accessory sequences, and plasmid sequences.
